# The predictive value of serum 25(OH)D, AQP4, and IL-4 levels for edema volume and clinical prognosis after traumatic brain injury

**DOI:** 10.3389/fmed.2026.1788253

**Published:** 2026-04-21

**Authors:** Yuanpeng Wang, Zhiying Ding, Lixiang Yang, Yunna Tao, Yanhong Song

**Affiliations:** 1 Department of Clinical Laboratory, The 904th Hospital of Joint Logistic Support Force (Wuxi Taihu Hospital), Wuxi, China; 2 Department of Neurosurgery, The Affiliated Wuxi People’s Hospital of Nanjing Medical University, Wuxi People’s Hospital, Wuxi Medical Center, Nanjing Medical University, Wuxi, Jiangsu, China; 3 Department of Neurosurgery, The 904th Hospital of Joint Logistic Support Force (Wuxi Taihu Hospital), Wuxi, China; 4 Department of Orthopedics, The 904th Hospital of Joint Logistic Support Force (Wuxi Taihu Hospital), Wuxi, China

**Keywords:** 25-hydroxyvitamin D, AQP4, brain edema volume, interleukin-4, outcome, traumatic brain injury

## Abstract

**Objective:**

This study aims to explore the associations between serum 25-hydroxyvitamin D (25(OH)D), aquaporin 4 (AQP4), and interleukin-4 (IL-4) levels with brain edema volume and outcomes in patients with traumatic brain injury (TBI).

**Methods:**

A cohort of 279 TBI patients, enrolled between January 2023 and December 2024, was analyzed. Serum levels of 25(OH)D, AQP4, and IL-4, as well as brain edema volume, were measured on days 1, 3, and 7 post-admission. Pearson correlation analysis was used to assess relationships between these serum markers and brain edema volume. Based on the 90-day follow-up outcome, patients were classified into a favorable outcome group (*n* = 215) and a poor outcome group (*n* = 64). Multivariate logistic regression was employed to identify factors influencing TBI outcomes.

**Results:**

Serum 25(OH)D levels were highest on day 1 post-TBI, followed by day 3 and day 7 (*P* < 0.05). In contrast, AQP4 and IL-4 levels, along with brain edema volume, were lowest on day 1 and increased significantly by days 3 and 7 (*P* < 0.05). Serum 25(OH)D levels were inversely correlated with brain edema volume, while AQP4 and IL-4 levels exhibited a positive correlation (*P* < 0.05). On days 1, 3, and 7, the favorable outcome group had higher serum 25(OH)D levels and lower AQP4 and IL-4 levels compared to the poor outcome group (*P* < 0.05). Multivariate analysis revealed that serum 25(OH)D on day 1 and the Glasgow Coma Scale (GCS) score were associated with favorable outcomes, whereas IL-4 levels on day 1 independently predicted poor prognosis (*P* < 0.05). ROC curve analysis demonstrated that all tested biomarkers had prognostic value, with serum 25(OH)D on day 1 showing the highest predictive accuracy.

**Conclusion:**

Serum levels of 25(OH)D, AQP4, and IL-4 are significantly associated with brain edema volume in TBI patients. Among these markers, serum 25(OH)D levels on the first day post-injury serve as the most reliable prognostic indicator of patient outcomes.

## Introduction

Traumatic brain injury (TBI) is a leading cause of death and disability globally, attracting significant attention due to its epidemiological characteristics and economic burden both worldwide and in China (1). According to the Global Burden of Disease study, 1,243,068 deaths from road traffic accidents were attributed to TBI in 2017. Despite improvements in mortality rates from road injuries, the incidence continues to rise, with notable geographical disparities (2). In China, TBI incidence and mortality rates exhibit similar trends, particularly among the elderly, where both have significantly increased (3). TBI not only severely affects individual health but also imposes a substantial economic burden. Research indicates that treatment and rehabilitation costs for TBI patients are high, especially in low- and middle-income countries, where the burden is most pronounced (4). In China, the treatment costs and long-term care needs for TBI patients place considerable strain on the healthcare system and social resources. Moreover, the long-term consequences of TBI, such as persistent neurovascular dysfunction, may lead to the development of late-onset neurodegenerative diseases, further intensifying social and economic burdens (5).

Hematoma volume and the severity of cerebral edema are key factors influencing intracranial pressure changes and patient outcomes. Studies have shown that edema progression correlates strongly with increased intracranial pressure and poor functional outcomes (6, 7). Worsening cerebral edema can result in neurological deterioration or even brain herniation. Are there serum biomarkers capable of predicting hematoma expansion, the extent of brain edema, and patient prognosis? Recent research highlights inflammation as a key factor in the formation of brain edema and hematoma expansion following TBI (8, 9). As biomarker research advances, numerous inflammatory factors have been identified as potential contributors to the inflammatory response and brain edema after TBI (10–12).

25-Hydroxyvitamin D [25(OH)D] is the principal circulating form of vitamin D in the body, known for its antioxidative and anti-inflammatory properties. A multicenter observational study revealed that TBI patients with vitamin D deficiency exhibited poorer functional recovery at discharge and significantly lower 6-month survival rates (13). Aquaporin 4 (AQP4), a water channel protein predominantly expressed in astrocytes, plays a pivotal role in maintaining intracranial fluid balance (14). Post-TBI, significant changes in AQP4 expression and localization are strongly linked to the development of brain edema (15). Additionally, research indicates that sleep disruption exacerbates post-TBI neuroinflammatory responses and correlates with AQP4 polarization abnormalities, further emphasizing the pivotal role of AQP4 in post-TBI inflammatory processes (16). Interleukin-4 (IL-4) plays a critical role in TBI pathogenesis, particularly in regulating neuroinflammation and promoting neural repair (17, 18). As an anti-inflammatory cytokine, IL-4 reduces inflammatory responses by promoting the M2 phenotype transition in macrophages and microglia, which not only limits neuronal damage but also enhances neural regeneration and restores white matter integrity (19, 20). Therefore, 25(OH)D, AQP4, and IL-4 hold substantial therapeutic potential for TBI treatment. However, most of the available research is derived from animal or cell studies, with a lack of large-scale clinical trials for validation. This study aims to explore the relationships between serum 25(OH)D, AQP4, and IL-4 levels and edema volume and outcomes in TBI patients.

## Materials and methods

### Study design

A retrospective study was conducted at Wuxi Taihu Hospital, Jiangsu, China, from January 2023 to December 2024. During this period, 718 patients with acute TBI were screened, and 279 patients who underwent 25(OH)D, AQP4, and IL-4 detection and had complete clinical data were initially enrolled. The study protocol was approved by the Wuxi Taihu Hospital Clinical Research Ethics Committee (2025-YXLL-019) and adhered to the principles of the Declaration of Helsinki. Informed consent was waived as the study was retrospective.

### Patients enrolled in the study and the sample selection procedures

Patients included in the study underwent neurosurgery at our hospital. The inclusion criteria were as follows: (1) aged 18–75 years; (2) diagnosed with TBI based on cranial CT findings, including cerebral subdural hematoma, cerebral epidural hematoma, intracerebral hematoma, subarachnoid hemorrhage, or cerebral contusion; (3) Glasgow Coma Scale (GCS) score of 3–12; (4) time from trauma to admission < 24 h; and (5) availability of complete clinical and follow-up data. Exclusion criteria included: (1) incomplete imaging data; (2) age under 18 years or over 75 years; (3) severe dysfunction of the heart, lungs, liver, or other organs; (4) malignant tumors; (5) nontraumatic cerebral hemorrhage; (6) recent use of hormones or immunosuppressants; (7) tuberculosis or autoimmune diseases; and (8) severe multiple trauma.

### Clinical data collection

General data were collected from the 279 TBI patients, including age, sex, BMI, hemorrhage location, smoking history, alcohol consumption history, admission brain edema volume, presence of hypertension, diabetes, coronary heart disease, systolic and diastolic blood pressure, and admission GCS score.

### Detection of serum 25(OH)D, AQP4, and IL-4 levels

On days 1, 3, and 7 after cerebral hemorrhage, 5 mL of peripheral venous blood was collected from each patient under fasting conditions. The samples were centrifuged at 3,500 rpm for 10 min with a centrifugation radius of 8 cm. Serum was separated and stored at −80°C for later analysis. Serum levels of 25(OH)D (Shanghai Zhuocai Biotechnology Co., Ltd.), AQP4 (Shanghai Jingkang Bioengineering Co., Ltd.), and IL-4 (Shanghai Hudin Biotechnology Co., Ltd.) were measured using enzyme-linked immunosorbent assay (ELISA) kits. All procedures were performed according to the manufacturers’ instructions.

### Assessment of brain edema and hematoma volume

Cranial CT examinations were conducted on days 1, 3, and 7 after TBI. CT perfusion imaging technology was used to analyze the edematous areas of the brain, with the edema and hematoma volumes measured at each layer. The degree of brain edema and hematoma volume were calculated using the Tada formula: the product of the long axis, short axis, slice thickness, number of slices, and π/6 for hematoma volume. The total lesion volume was the sum of the hematoma/high-density lesion volume and the total volume of the edematous area. The degree of brain edema and hematoma volume was calculated as the difference between the total lesion volume and the high-density lesion volume.

### Outcome assessment and grouping

All patients received standardized treatment in accordance with established treatment guidelines (21, 22). Based on 90-day follow-up data, TBI patients were categorized into a favorable outcome group and a poor outcome group according to the Glasgow Outcome Scale (GOS) score (23). A favorable outcome was defined as a GOS score of 4 or 5, while a poor outcome was defined as a GOS score of 1–3.

### Statistical analysis

Data were analyzed using SPSS 25.0 statistical software. Measurement data are expressed as mean ± standard deviation ($\bar{x}$ ± s). Comparisons between two groups were conducted using independent samples *t*-tests, while comparisons among multiple groups were performed using one-way ANOVA, with pairwise comparisons within groups carried out using paired samples *t*-tests. Categorical data are presented as n (%) and analyzed using the χ^2^ test. Pearson correlation analysis was employed to explore the relationships between serum 25(OH)D, AQP4, and IL-4 levels and brain edema volume. Multivariate logistic regression analysis was applied to identify influencing factors. Receiver operating characteristic (ROC) curves were constructed to assess the predictive value of serum 25(OH)D, AQP4, and IL-4 levels, along with brain edema volume, for TBI prognosis. A *P*-value of < 0.05 was considered statistically significant.

## Results

### Comparison of serum 25(OH)D, AQP4, and IL-4 levels and the brain edema volume in TBI patients at different time points

**FIGURE 1:**
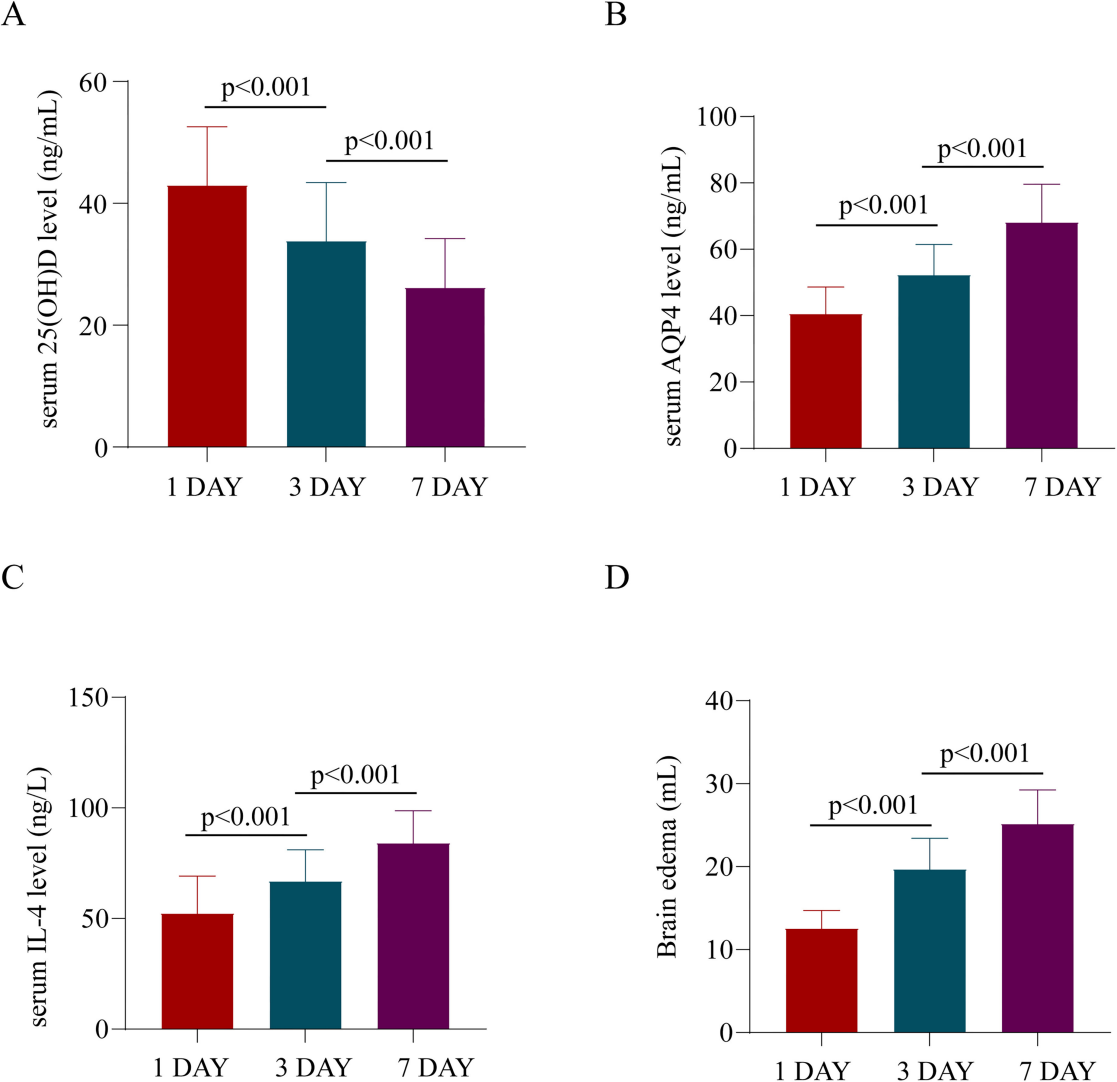
Comparison of serum 25(OH)D, AQP4, and IL-4 levels and brain edema volume in TBI patients at different time points. **(A)** Serum 25(OH)D levels. **(B)** Serum AQP4 levels. **(C)** Serum IL-4 levels. **(D)** Brain edema volume. *P* < 0.001.

In TBI patients, the serum 25 (OH)D levels followed this pattern: day 1 after TBI > day 3 > day 7; the IL-4 levels were as follows: day 1 < day 3 < day 7; and the AQP4 levels, hematoma volume, and brain edema volume showed the pattern: day 1 < day 3 < day 7. The differences were statistically significant (*P* < 0.05, Figure 1).

### Relationships between serum 25(OH)D, AQP4, and IL-4 levels and brain edema volume in TBI patients

**FIGURE 2:**
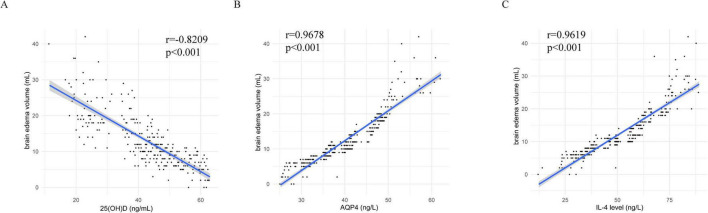
Relationships between serum 25(OH)D, AQP4, and IL-4 levels and brain edema volume in TBI patients. **(A)** The relationship between serum 25(OH)D and brain edema. **(B)** The relationship between serum AQP4 and brain edema. **(C)** The relationship between serum IL-4 and brain edema. *P* < 0.001.

To investigate the relationships between serum 25(OH)D, AQP4, and IL-4 levels and brain edema volume in TBI patients, biomarkers and brain edema volume were measured on day 1 post-TBI. A negative correlation was observed between serum 25 (OH)D levels and brain edema volume, while both AQP4 and IL-4 levels were positively correlated with brain edema volume (*P* < 0.05, Figure 2).

### Comparisons of serum 25(OH)D, AQP4, and IL-4 levels at different time points in TBI patients with different outcomes

**FIGURE 3:**
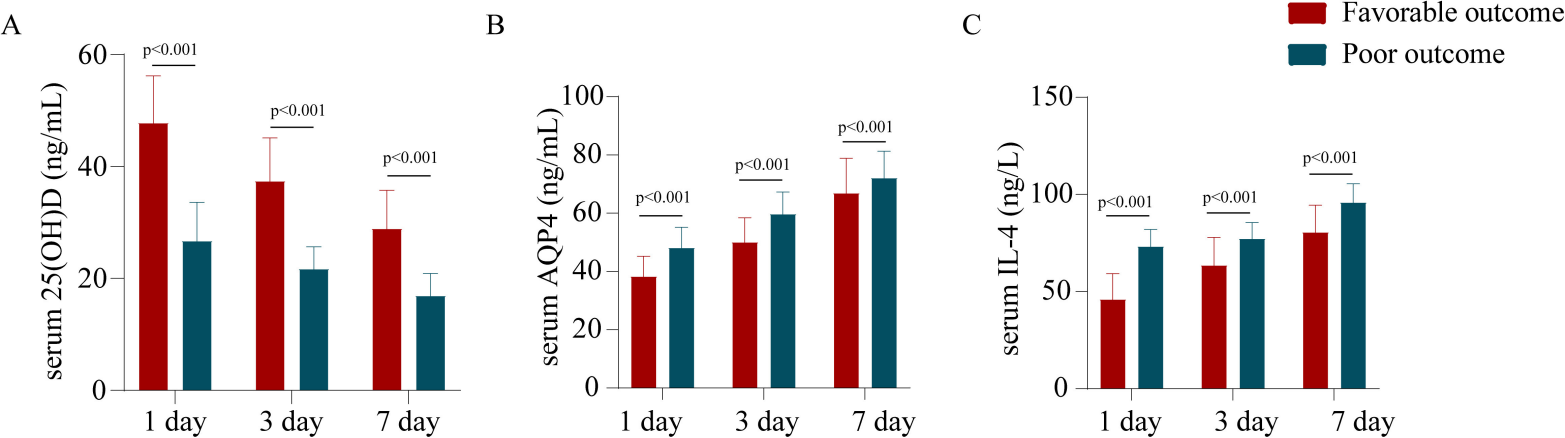
Comparison of serum 25(OH)D, AQP4, and IL-4 levels at different time points in TBI patients with different outcomes. **(A)** Serum 25(OH)D levels in different prognosis groups. **(B)** Serum AQP4 levels in different prognosis groups. **(C)** Serum IL-4 levels in different prognosis groups.

To assess the impact of serum biomarkers on prognosis, patients were categorized into a good prognosis group and a poor prognosis group based on the GOS score. The serum 25(OH)D levels on days 1, 3, and 7 post-TBI were higher in the favorable prognosis group compared to the poor prognosis group, whereas AQP4 and IL-4 levels were significantly lower in the favorable prognosis group (*P* < 0.001, Figure 3).

### Univariate analysis of disease outcomes in TBI patients

No statistically significant differences were observed between the two groups in terms of age, sex, BMI, hemorrhage location, smoking history, drinking history, presence of hypertension, diabetes, coronary heart disease, systolic or diastolic blood pressure (*P* > 0.05). However, the admission brain edema volume and GCS score were significantly higher in the poor prognosis group compared to the favorable prognosis group (*P* < 0.05, Table 1).

**TABLE 1:** Univariate analysis of outcomes in TBI patients [n (%), ($\bar{x}$ ± s)].

	Survival Group (*n* = 215)	Death Group (*n* = 64)	t/χ^2^/Z-value	*P*-value
Gender			0.242	0.665
Male	127 (59.07)	40 (62.50)		
Female	88 (40.93)	24 (37.50)		
Age (years)	46.75 ± 8.33	45.89 ± 7.92	0.733	0.464
BMI (kg/m^2^)	22.81 ± 2.52	23.01 ± 2.93	0.536	0.592
Admission brain edema vol (mL)	8.98 ± 2.07	15.36 ± 2.42	19.53	< 0.001
Hemorrhage location			−0.159	0.875
Epidural	48 (22.33)	13 (20.31)		
Subdural	75 (34.88)	23 (35.94)		
Subarachnoid	56 (26.05)	18 (28.13)		
Intraparenchymal	36 (16.74)	10 (15.62)		
Smoking history	67 (31.16)	20 (31.25)	0	1.000
Drinking history	41 (34.75)	15 (44.12)	0.586	0.478
Hypertension	45 (20.93)	17 (26.56)	0.905	0.392
Diabetes	38 (17.67)	12 (18.75)	0.039	0.854
Coronary heart disease	21 (9.77)	9 (14.06)	0.948	0.359
Systolic BP (mmHg)	89.93 ± 11.62	91.75 ± 12.28	1.086	0.279
Diastolic BP (mmHg)	119.41 ± 19.88	117.95 ± 20.16	0.514	0.608
Admission GCS score	9.27 ± 2.04	6.19 ± 1.71	10.98	<0.001

### Multivariate logistic regression analysis of factors affecting the prognosis of TBI patients

Variables with statistically significant differences in the univariate analysis (admission brain edema volume, admission GCS score, and serum 25 (OH)D, AQP4, and IL-4 levels on day 1) were selected as independent variables, with prognosis (poor outcome = 1, favorable outcome = 0) as the dependent variable. These variables were included in a multivariate logistic regression model. The results showed that serum 25 (OH)D levels, IL-4 levels, and the GCS score were significant factors influencing the prognosis of TBI patients. Specifically, serum 25(OH)D levels and the GCS score were protective factors, while IL-4 levels were an independent risk factor (*P* < 0.05, Table 2).

**TABLE 2:** Multivariate logistic regression analysis of factors affecting the prognosis of TBI patients.

	β value	SE value	Wald χ^2^-value	*P*-value	OR, 95% CI
Brain edema	−0.159	0.172	0.858	0.354	0853 (0.609, 1.194)
GCS Score	−0.685	0.191	12.877	<0.001	0.504 (0.347, 0.733)
Serum 25(OH)D	−0.334	0.071	22.229	<0.001	0.716 (0.623, 0.823)
Serum AQP4	−0.297	0.256	1.348	0.246	0.743 (0.450, 1.227)
Serum IL-4	0.199	0.066	9.178	0.002	1.220 (1.073, 1.388)

### The predictive value of admission brain edema score, admission GCS score, and serum 25(OH)D, AQP4, and IL-4 levels on day 1 for the prognosis of TBI patients

**FIGURE 4:**
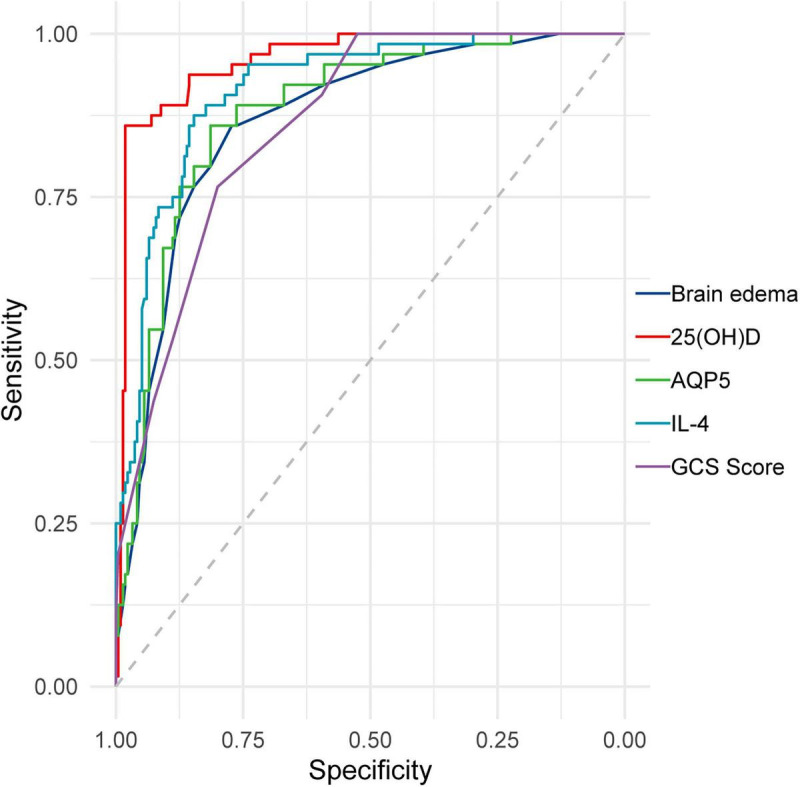
ROC curves of serum 25(OH)D, AQP4, and IL-4 levels; brain edema; and the GCS score for predicting the prognosis of TBI patients.

The areas under the ROC curves (95% CIs) for predicting TBI prognosis based on admission brain edema, admission GCS score, and serum 25(OH)D, AQP4, and IL-4 levels on day 1 were 0.868 (0.8195, 0.9159), 0.862 (0.8179, 0.9052), 0.959 (0.9348, 0.9839), 0.881 (0.836, 0.9261), and 0.915 (0.8783, 0.952), respectively. The sensitivities and specificities are shown in Figure 4. Although all of these indicators demonstrated predictive value for patient prognosis, serum 25(OH)D exhibited superior predictive ability compared to admission brain edema, admission GCS score, and AQP4 and IL-4 levels on day 1 (Figure 4).

## Discussion

TBI is typically caused by external forces, with initial symptoms such as vomiting and headache. As the condition progresses, the accumulation of inflammatory responses and brain edema can lead to more severe symptoms, including consciousness disturbances and limb paralysis, complicating treatment and adversely affecting prognosis. Identifying biomarkers that can predict the degree of brain edema early and provide prognostic insights is critical for improving disease outcomes (24–26). In this study, the levels of biomarkers such as 25(OH)D, AQP4, and IL-4, as well as brain edema volume, varied at different time points after TBI and were closely positively correlated with the severity of the patient’s condition. Specifically, serum 25(OH)D levels were negatively correlated with brain edema volume, whereas AQP4 and IL-4 levels were positively correlated with brain edema volume in TBI patients. Serum 25(OH)D, IL-4 levels, and the GCS score were identified as factors influencing the prognosis of TBI patients and exhibited predictive value for patient outcomes.

Recent studies have highlighted the significant role of 25(OH)D in the pathophysiology of brain diseases (27, 28). Zhang et al. (29) reported a thirty-five TBI patients study, it showed that no significant correlation between the overall plasma level of 25(OH)D and cognitive function in TBI patients, but cognitive impairment was more common when 25(OH)D levels ranged from 10 to 30 ng/ml. The negative results of this study are attributed to the extremely small sample size and the overly long detection time window. Previous studies have mostly focused on the potential correlation between vitamin D deficiency and the poor prognosis of TBI patients (13, 30). However, there have been few studies that directly examined the relationship between serum vitamin D/25(OH)D levels and the prognosis of TBI patients. To our data, this is a large-scale study. In the present study, serum 25(OH)D levels gradually decreased over time following TBI and showed a negative correlation with brain edema volume. Additionally, the serum 25(OH)D levels on days 1, 3, and 7 post-TBI were higher in the favorable prognosis group compared to the poor prognosis group, further supporting the role of 25(OH)D in brain edema and TBI prognosis. Several factors may explain these findings: After TBI, brain tissue damage triggers an inflammatory response, activating and releasing numerous inflammatory cells and mediators, which contribute to the formation and worsening of brain edema. This inflammatory process inhibits vitamin D metabolism, leading to a decrease in the active form of vitamin D, 25(OH)D. A reduction in 25(OH)D levels, in turn, exacerbates the inflammatory response, negatively impacting prognosis.

AQP4 plays a pivotal role in TBI and subsequent cerebral edema. AQP4 is a water channel protein primarily localized on the foot processes of astrocytes, responsible for regulating water flow and homeostasis in the brain. In TBI, significant alterations in AQP4 expression and distribution are closely linked to the development of cerebral edema. A retrospectively study examined brain samples in 145 cases of death after different survival times following TBI, it showed brain AQP4 was upregulated after TBI, While the elationship between the expression level and prognosis is unclear (31). Research has demonstrated that targeting AQP4 expression can effectively alleviate TBI-induced cerebral edema, thus improving patient prognosis (15, 32). The level of AQP4 in cerebrospinal fluid (CSF) has also been shown to correlate with the severity and prognosis of TBI (33). In one study, AQP4 levels peaked on day 14 post-hospitalization and significantly decreased by day 28. Higher AQP4 levels were associated with poorer outcomes, suggesting that AQP4 may serve as a potential biomarker for assessing TBI severity and predicting outcomes (33). Furthermore, AQP4 expression is tightly linked to the development of cerebral edema. After TBI, increased AQP4 expression exacerbates cytotoxic edema (8). Activation of the Foxo3a transcription factor induces cerebral edema by upregulating AQP4 levels, a mechanism confirmed in a controlled cortical impact model in mice (8). In the present study, AQP4 levels were closely associated with disease severity and cerebral edema, reinforcing AQP4’s significant role in the pathophysiology of TBI-induced cerebral edema. Monitoring AQP4 levels can enhance prognostic accuracy, guide therapeutic interventions, and improve clinical decision-making. These findings strongly support AQP4 as a promising therapeutic target in TBI.

Excessively high levels of IL-4 can regulate inflammatory responses and impact disease progression. Eftekharian (34) reported that serum IL-4 levels are elevated in patients with schizophrenia and correlate with disease progression. Other clinical studies have shown that IL-4 is closely related to the prognosis of TBI patients and cerebral edema (35, 36). In this study, serum IL-4 levels increased over time following TBI, and these levels were positively correlated with brain edema volume. Additionally, serum IL-4 levels on days 1, 3, and 7 after TBI were lower in the favorable outcome group compared to the poor outcome group. The increase in IL-4 promotes the release of inflammatory mediators, exacerbating the inflammatory response, which in turn increases brain edema volume and negatively impacts prognosis.

However, this study has several limitations, such as its small sample size and single-center design, which may introduce bias. Future research should involve large-scale, multicenter studies with multiple observation time points to further validate the combined predictive value of these factors for TBI prognosis.

In conclusion, serum 25(OH)D concentration, AQP4 concentration, IL-4 concentration, brain edema volume, and GCS score are key factors influencing the prognosis of TBI patients. Among these, serum 25(OH)D appears to have significant predictive value for patient outcomes.

## Data Availability

The raw data supporting the conclusions of this article will be made available by the authors, without undue reservation.
